# Identifying hub genes of clear cell renal cell carcinoma associated with the proportion of regulatory T cells by weighted gene co-expression network analysis

**DOI:** 10.18632/aging.102397

**Published:** 2019-10-31

**Authors:** Ye-Hui Chen, Shao-Hao Chen, Jian Hou, Zhi-Bin Ke, Yu-Peng Wu, Ting-Ting Lin, Yong Wei, Xue-Yi Xue, Qing-Shui Zheng, Jin-Bei Huang, Ning Xu

**Affiliations:** 1 Department of Urology, The First Affiliated Hospital of Fujian Medical University, Fuzhou 350005, China

**Keywords:** ccRCC, weighted gene co-expression network analysis, Treg cells, hub genes, anti-tumor immune

## Abstract

Background: Numerous patients with clear cell renal cell carcinoma (ccRCC) experience drug resistance after immunotherapy. Regulatory T (Treg) cells may work as a suppressor for anti-tumor immune response.

Purpose: We performed bioinformatics analysis to better understand the role of Treg cells in ccRCC.

Results: Module 10 revealed the most relevance with Treg cells. Functional annotation showed that biological processes and pathways were mainly related to activation of the immune system and the processes of immunoreaction. Four hub genes were selected: LCK, MAP4K1, SLAMF6, and RHOH. Further validation showed that the four hub genes well-distinguished tumor and normal tissues and were good prognostic biomarkers for ccRCC.

Conclusion: The identified hub genes facilitate our knowledge of the underlying molecular mechanism of how Treg cells affect ccRCC in anti-tumor immune therapy.

Methods: The CIBERSORT algorithm was performed to evaluate tumor-infiltrating immune cells based on the Cancer Genome Atlas cohort. Weighted gene co-expression network analysis was conducted to explore the modules related to Treg cells. Gene Ontology analysis and pathway enrichment analysis were performed for functional annotation and a protein–protein interaction network was built. Samples from the International Cancer Genomics Consortium database was used as a validation set.

## INTRODUCTION

Renal cell carcinoma (RCC) is one of the most common urological malignant tumors, accounting for approximately 400,000 new cases and 175,000 deaths worldwide in 2018 [1], which is a heavy burden on health care systems. Clear cell renal cell carcinoma (ccRCC) comprises the most common RCC histological subtype [2]. ccRCC initially presents as metastasis in 30% patients, and up to 40% patients undergoing surgical excision develop local recurrence or metastatic disease [3]. Although immunotherapy has shown remarkable success in ccRCC, a part of patients experience drug resistance and disease progression after treatment, for which individual variation at the genetic level may be responsible [4–6]. Additionally, tumor-infiltrating immune cells and the tumor microenvironment are thought to be relevant to this [7, 8].

Regulatory T (Treg) cells characterized by expression of the master regulatory transcription factor FOXP3 are a highly immune-suppressive subset of CD4+ T cells that maintain immune homeostasis [9]. They act as a gatekeeper of almost any type of immune reaction. On one hand, they can suppress unwanted immune responses such as autoimmunity, allergy, or transplant rejection. On the other hand, they can also prevent protective immune responses against invading pathogens or tumors, and exert unproductive immunosuppression leading to unwanted reactions or even promote disease progression [10]. Kamada et al. reported that proliferation of Treg cells induced by PD-1 blockade results in the inhibition of antitumor immunity, which reduces the effect of anti-PD-1 [11]. Liotta et al. reported that Treg cells are associated with poor prognosis in renal cancer [12]. Therefore, targeting Treg cells should be crucial to improving the treatment outcomes of cancer immunotherapy. Previous studies have reported several genetic biomarkers for the prognosis of ccRCC, however, relationships between Treg cells and these biomarkers, and how Treg cells cause drug resistance and poor prognosis still remain unknown [13–15].

Development of microarray and sequencing technology provides an excellent tool and platform for cancer research. By associating clinical data with molecular mechanisms, new biomarkers for diagnosis, treatment, and prognosis may be discovered. Weighted gene co-expression network analysis (WGCNA) is an algorithm for weighted correlation network analysis and can be used as a data exploratory tool or a gene screening method to identify clusters of highly correlated genes [16]. It is has been widely used to determine hub genes in various cancers. To further explore the mechanism through which Treg cells cause poor prognosis in ccRCC, we used this algorithm to identify relevant modules and hub genes.

## RESULTS

### Tumor-infiltrating Treg cells in ccRCC

**Figure 1:**
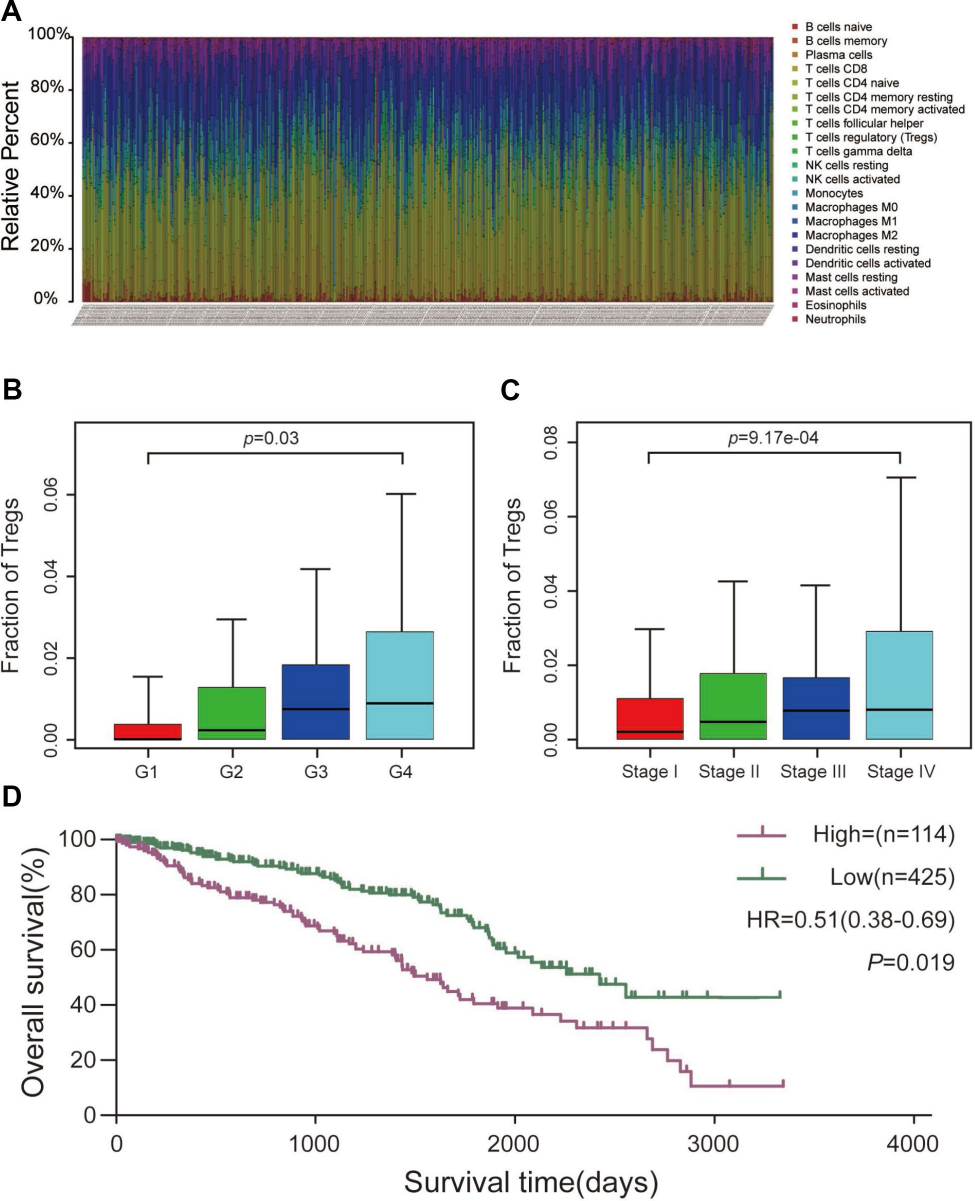
**CIBERSORT analysis and clinical significance of Treg cells in ccRCC**. (**A**) Relative percentage of each type of immune cell in 539 ccRCC samples from TCGA cohort. (**B**) Proportion of Treg cells in different pathological grades. (**C**) Proportion of Treg cells in different AJCC stages. (**D**) Overall survival between patients with high and low proportions of infiltrating Treg cells.

Using the CIBERSORT algorithm, we investigated the 22 subpopulations of infiltrating immune cells. Figure 1A summarizes the results obtained from 539 ccRCC samples. Data relating to age, gender, pathological grade, American Joint Committee on Cancer (AJCC) stage, and survival state were collected and are summarized in Table 1. The results showed that a higher proportion of Treg cells was associated with a higher pathological grade and a more advanced AJCC stage (Figure 1B and 1C). Additionally, patients with a high proportion of infiltrating Treg cells had a poorer prognosis than those with a low proportion (Figure 1D).

**Table 1:** Clinicopathological characteristics of 539 patients with ccRCC from TCGA.

**Variables**	**Fraction of Tregs**			
	**N**	**Low**	**High**	***P*-value**
Total, n(%)	539	425	114	
Age				0.857
<60y	256	201	55	
≥60y	283	224	59	
Gender				0.584
Male	350	273	77	
Female	189	152	37	
AJCC stage				0.001*
I	258	219	39	
II	68	49	19	
III	132	104	28	
IV	81	53	28	
Pathological grade				0.011*
G1	12	10	2	
G2	216	182	34	
G3	220	172	48	
G4	91	61	30	
Survival				0.001*
Yes	360	302	58	
No	179	123	56	

* *P* <0.05.

### DEGs screening

After conducting the CIBERSORT analysis, we obtained the proportion of Treg cells and the expression data of 432 ccRCC samples. Cases were divided into two groups (114 cases with a high proportion of Treg cells and 425 cases with a low proportion of Treg cells) with a cut-off value that was the mean value of the proportion of Treg cells. Under the threshold of adjusted *P*-value <0.05 and |logFC|≥2, a total of 4,921 DEGs (2,348 upregulated and 2,573 downregulated) were chosen for subsequent analysis.

### Weighted co-expression network construction and key module identification

**Figure 2:**
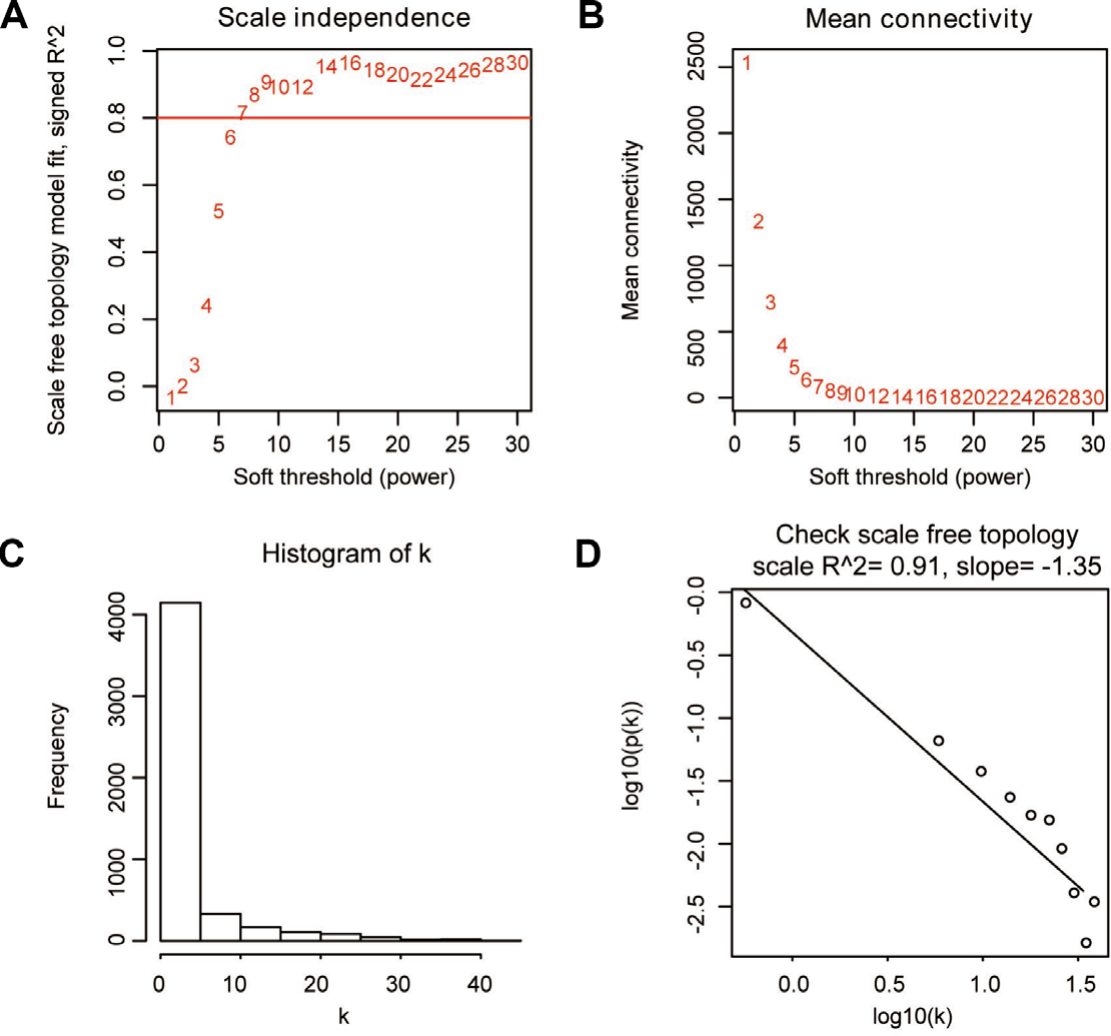
**Determination of soft-thresholding parameter in WGCNA.** (**A**) Analysis of the scale-free fit index for various soft-thresholding parameters. (**B**) Analysis of the mean connectivity for various soft-thresholding parameters. (**C**) Histogram of connectivity distribution when β=9. (**D**) Check of scale-free topology when β=9.

**Figure 3:**
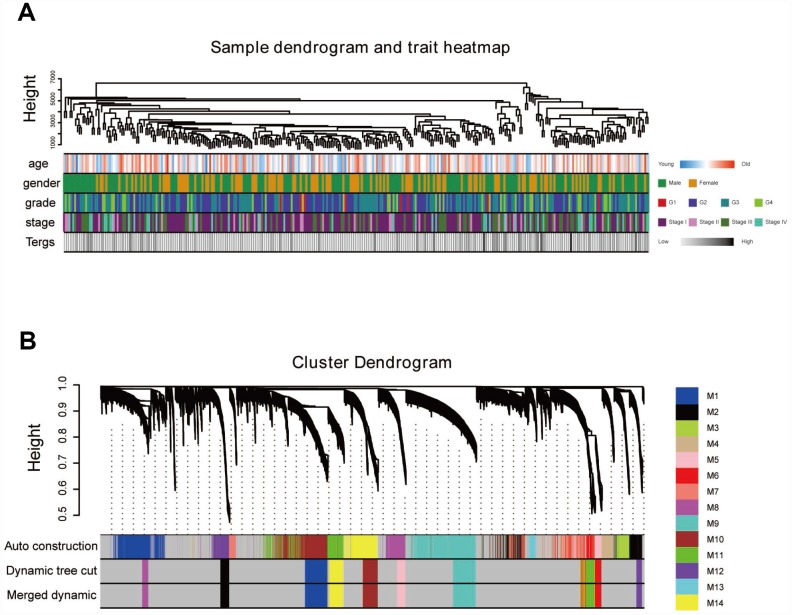
**Sample dendrogram and clustering dendrogram of WGCNA.** (**A**) Sample dendrogram and corresponding clinical characteristics. (**B**) Cluster dendrogram of 432 samples with eligible data.

**Figure 4:**
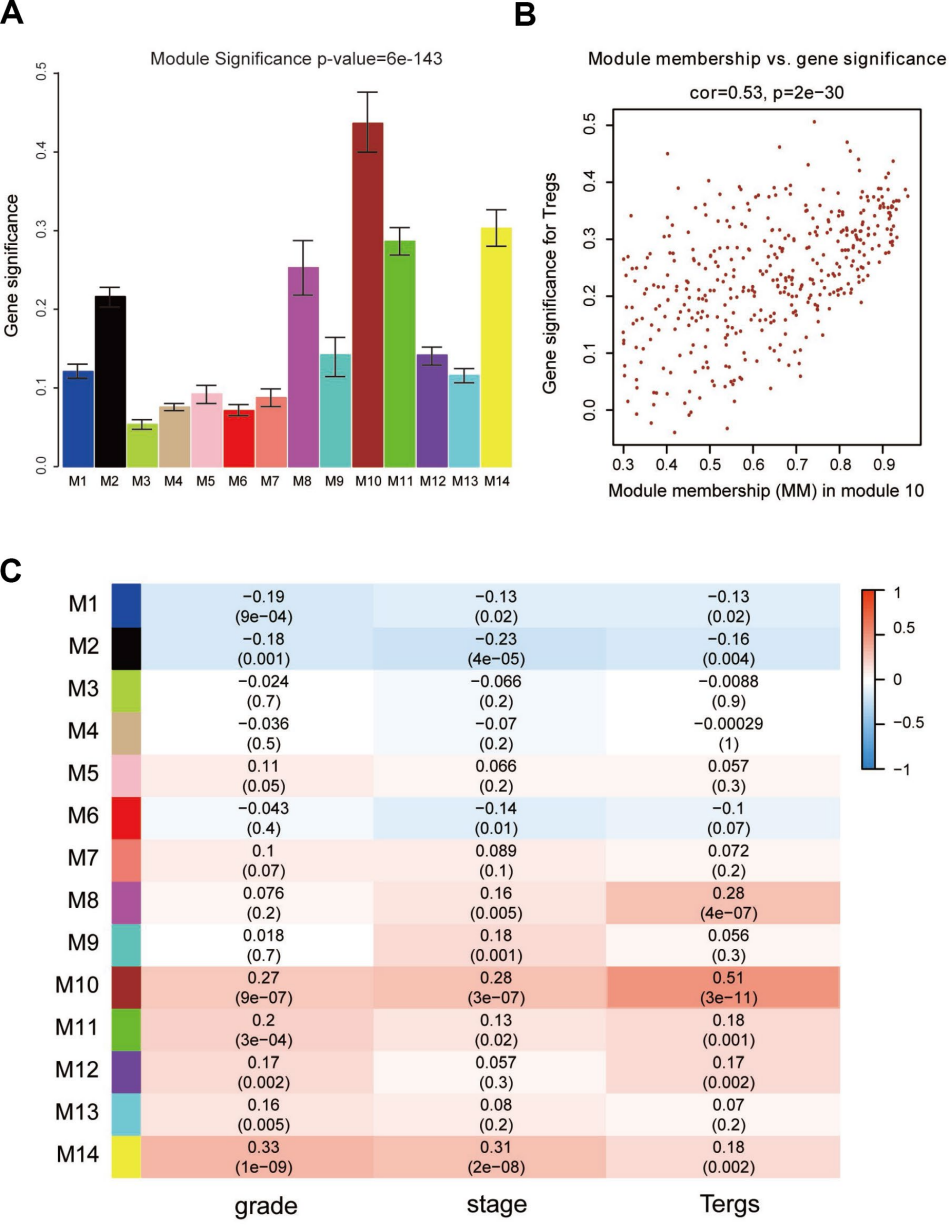
**Identification of modules associated with clinical characteristics.** (**A**) Distribution of average gene significance and errors in the modules associated with the proportion of Treg cells in ccRCC. (**B**) Scatter plot of module eigengenes in module 10. (**C**) Heatmap of the correlation between module eigengenes and different clinical characteristics of ccRCC.

“WGCNA” R package was used to categorize the DEGs with similar expression patterns into modules by average linkage clustering, based on the 432 cases with eligible CIBERSORT data. Firstly, we selected the power of β=9 (scale free R^2^=0.91) as the soft-thresholding parameter (Figure 2A, 2B); Figure 2C and 2D shows the positive result of the rationality test. After that, a sample dendrogram was constructed based on the similarity between the samples and the clinical characteristics of each sample are shown (Figure 3A). Finally, fourteen modules were identified (Figure 3B). We used two methods to test the relevance between each module and the fraction of Treg cells. Modules with a greater MS were considered to have more connection with Treg cells, and we found that the MS of module 10 was higher than any other module (Figure 4A). Afterwards, the ME of module 10 showed a higher correlation with the fraction of Treg cells than other modules (Figure 4C). Based on the two methods, we finally identified module 10 was the module most relevant to Treg cells in ccRCC (Figure 4B).

### GO and pathway enrichment analysis

**Figure 5:**
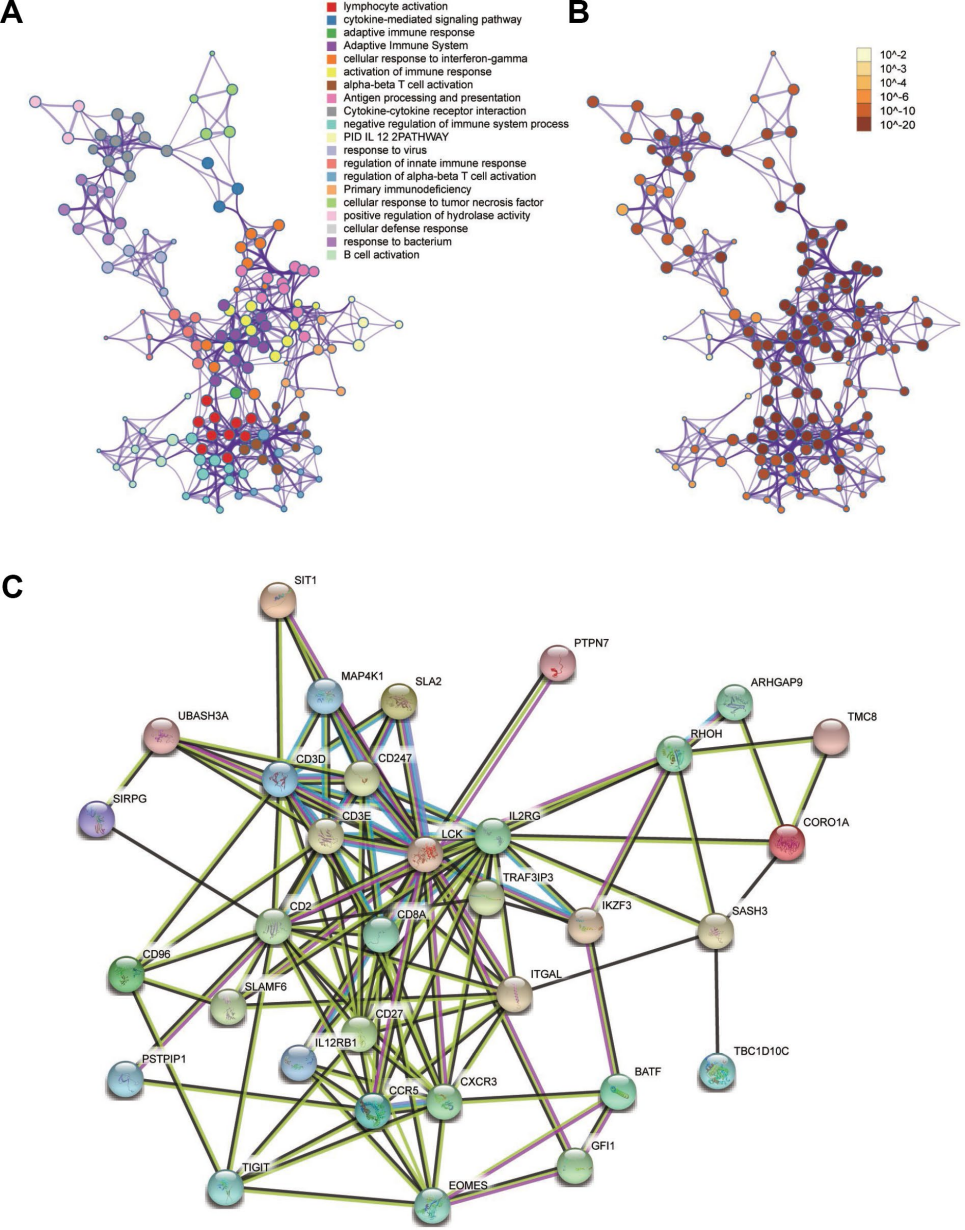
**Functional enrichment analysis and construction of PPI network.** (**A**) GO and pathway enrichment analysis of genes in the module 10. (**B**) *P*-value of each gene in the network. (**C**) PPI network constructed using STRING.

We performed a functional enrichment analysis to search for the biological processes and pathways relevant to the module 10 using Metascape. The results showed that the biological processes and pathways were mainly related to activation of the immune system and the processes of immunoreactions (Figure 5A and 5B).

### Identification of hub genes and analysis of modules from PPI networks

A PPI network was constructed using STRING (Figure 5C). The top four hub genes were selected according to the degree of connectivity. They were LCK (LCK proto-oncogene, Src family tyrosine kinase), MAP4K1 (mitogen-activated protein kinase kinase kinase kinase 1), SLAMF6 (SLAM family member 6), and RHOH (Ras homolog family member H).

### Validation and efficacy evaluation of hub genes

**Figure 6:**
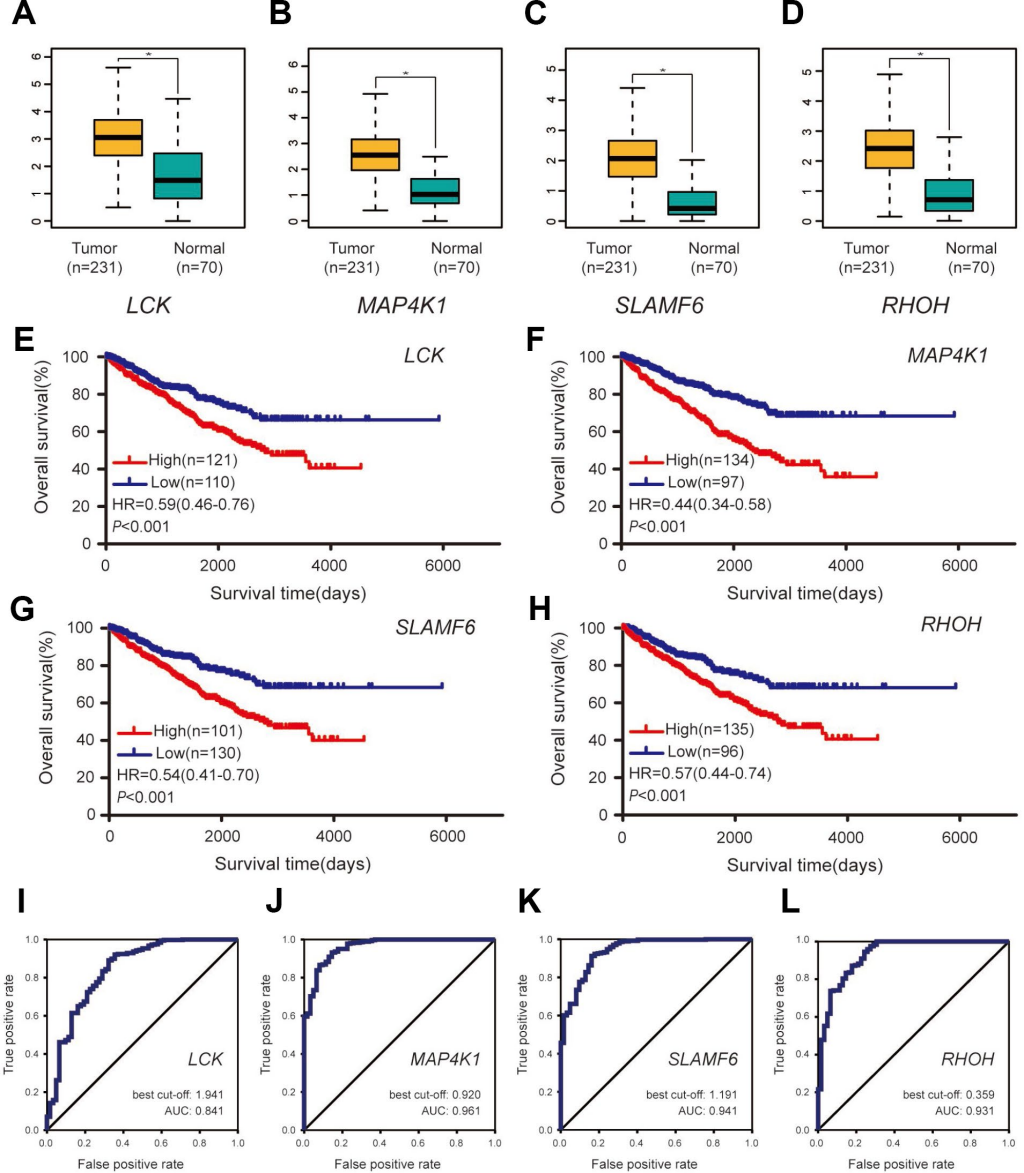
**Validation of the four hub genes based on the ICGC cohort.** (**A**–**D**) Expression levels of the four hub genes between ccRCC samples and normal tissues. (**E**–**H**) Overall survival between patients with high and low expression of the four hub genes. (**I**–**L**) ROC curves of the four genes to evaluate their capability in distinguishing tumor tissue and normal kidney tissue.

A dataset including 231 cases of ccRCC samples and 70 normal tissues from the ICGC database was used for validation. Compared with normal tissues, all four hub genes revealed higher expression levels in ccRCC samples (Figure 6A–6D). Survival analyses were performed grouped by the differential expression of the four hub genes. Table 2 shows the clinicopathological characteristics in patients with ccRCC from the ICGC cohort. It was found that increased expression levels of LCK (HR 0.59 [0.46–0.76], *P*<0.001) were associated with poor overall survival of ccRCC patients, as well as MAP4K1 (HR 0.44 [0.34–0.58], *P*<0.001), SLAMF6 (HR 0.54 [0.41–0.70], *P*<0.001), and RHOH (HR 0.57 [0.44–0.74], *P*<0.001) (Figure 6E–6H). In addition, ROC curve analyses were performed to evaluate the capability of the hub genes to distinguish tumor and normal tissues. AUC values for the four genes were greater than 0.5 (Figure 6I–6L).

**Table 2:** Clinicopathological characteristics of 231 patients with ccRCC from ICGC.

**Clinicopathological characteristics**	**Value**
Age, y	
Mean±SD	60.05±12.22
Range	26-90
Gender, n(%)	
Male	137(59.3)
Female	94(40.7)
AJCC stage, n(%)	
I	92(39.8)
II	60(26.0)
III	46(19.9)
IV	33(14.3)
Survival, n(%)	
Yes	107(46.3)
No	124(53.7)

## DISCUSSION

Unlike other cancers, advanced ccRCC responds poorly to chemotherapy and radiotherapy [17]. This has sparked further research on alternative therapies, most notably immunotherapy. In recent years, the landscape of management of advanced ccRCC has dramatically shifted with the development of immunotherapeutic agents, which are designed to repair, stimulate, and enhance the response of the immune system in attacking cancer cells [17].

Immune checkpoint blockade has shown remarkable efficacy and clinical application prospects in ccRCC; however, some patients have no response to the therapy, which can be explained by tumor immune escape [4–6]. Treg cells, a double-edged sword in human immune reactions, may play an important role in this. Takahiro et al reported that proliferation of Treg cells induced by PD-1 blockade results in inhibition of antitumor immunity, which reduces the effect of anti-PD-1 [11]. Nevertheless, how Treg cells decrease anti-tumor immunity and the related targets and pathways remain unclear. To explore how Treg cells affect ccRCC, we employed bioinformatics methods to identify relevant modules and hub genes. These findings could help to improve knowledge about the mechanism of immune escape and in the exploration of potential candidate genes or molecules for diagnosis, treatment, and prognosis.

In the present study, WCGNA was conducted to explore the modules and genes related to tumor-infiltrating Treg cells. The results showed that module 10 was the most relevant module. Additionally, functional annotation revealed that biological processes and pathways were mainly related to the activation of the immune system and the processes of immunoreactions, which was plausible and supported the result of WGCNA. Consequently, we explored the hub genes in module 10. Finally, LCK, MAP4K1, SLAMF6, and RHOH were selected from the PPI network according to the degree of connectivity. Follow-up validation showed that the four hub genes could well-distinguish tumor and normal tissues and were good prognostic biomarkers related to tumor-infiltrating Treg cells for ccRCC.

LCK, also known as lymphocyte-specific protein tyrosine kinase. is a member of the Src family of non-receptor protein tyrosine kinases [18]. It plays a vital role in various cellular processes such as cell cycle control, cell adhesion, motility, proliferation, and differentiation [18]. Physiologically, LCK is involved in the development, function, and differentiation of T cells. Existing evidence suggested that high expression of LCK was connected to the development and progression of tumors [19, 20]. A recent study reported that mutated and overexpressed LCK promoted the proliferation of acute myeloid leukemia cell lines [19]. Furthermore, LCK participates in many malignant biological processes in glioma, such as migration, tumor growth, and regulation of cancer stemness [20]. Qayyum et al. reported that LCK is a potential prognostic marker for renal cancer, which was consistent with this study, however, they did not explore the mechanism [21]. In castration-resistant prostate cancer (CRPC) mouse models, LCK nitrated and inactivated by myeloid-derived suppressor cell sensitizes CRPC mice to immune checkpoint blockade, which suggested that LCK acted as a potential target to improve the efficacy of immune checkpoint blockade [22]. Moreover, another study revealed that sorafenib could induce apoptosis, suppress cell activation, and cause cell cycle arrest in human peripheral blood T cells by targeting LCK phosphorylation, thus, supressing the immune reaction [23]. This presents a good approach for suppressing unwanted immune responses. Therefore, LCK is a promising therapeutic target for increasing response rates to immunotherapy.

MAP4K1, a member of the MAP4K family, is a hematopoietic-specific protein serine-threonine kinase. With its primary expression in hematopoietic cells, a potential regulatory role of MAP4K1 was suggested in mediating signaling of hematopoietic lineages [24]. Studies revealed the essential role of MAP4K1 in negatively regulating T cell activation with involvement of the linker of activated T cells and associated downstream signaling molecules [25, 26]. Consistently, studies also demonstrated that mice that received adoptive transfer of MAP4K1 knockout T cells became resistant to lung cancer growth via mounting effective anti-tumor immune responses, suggesting that inhibition of MAP4K1 could be a viable approach for cancer immune therapy by promoting the effector functions of T cells [27]. Liu et al. reported that inhibition of MAP4K1 will synergize with immune checkpoint modulator blockade as well as targets related to the prostaglandin E2 and adenosine pathways [26]. Accordingly, selective MAP4K1 inhibition was considered a means to enhance anti-tumor immunity. Sunitinib, a multi-receptor tyrosine kinase inhibitor approved for the targeted treatment of RCC, has recently been reported to inhibit the activation of the MAP4K1 protein in vitro [28]. Therefore, these findings may indicate a new target for sunitinib if confirmed by further studies.

SLAMF6 is a member of the SLAM family of receptors. SLAMF6 is usually expressed on a wide variety of immune cells including T cells, B cells, NK cells, double-positive thymocytes, eosinophils, and neutrophils. Previous studies revealed that SLAMF6 functions as an inhibitory receptor that controls autoimmunity in systemic lupus erythematosus [29]. Existing evidence suggested that this autoimmune suppression might also occur in anti-tumor immunity, which would reduce response rates to immune checkpoint blockade [30, 31]. However, further studies are required to confirm this.

RHOH is a hematopoietic-specific and GTPase-deficient member of the RHO-GTPase family. Its protein product is essential in the development of T lymphocytes. It plays an important role in many types of cancers, especially in cancers of myeloid [32]. Tajadura et al. reported that RHOH stimulated PC3 cell migration by promoting RAC1-driven membrane protrusion, resulting in a bad prognosis in prostate cancer [33]. Moreover, it was found that RHOH promoted the development of B cell chronic lymphocytic leukemia [34]. Furthermore, RHOH participates in a multi-protein complex with the zinc finger protein kaiso that regulates both cytoskeletal structures and chemokine-induced T cells [35]. Wang et al. reported that RHOH is a critical adapter protein, contributing to the regulation of both T cell receptor (TCR) and pre-TCR signalling during T cell development [36]. RHOH therefore acts as a crucial point in the regulatory pathways of immunoreactions and is a promising target for increasing the effectiveness of anti-tumor immune therapy. However, more studies are needed to confirm its value.

In summary, using a series of bioinformatics analyses, we identified four hub genes that were closely associated with the fraction of Treg cells in ccRCC. Our findings contribute more knowledge to the understanding of the underlying molecular mechanisms of how Treg cells affect ccRCC in anti-tumor immune therapy. However, further study is required to determine the exact mechanism in detail.

## MATERIALS AND METHODS

### Data collection and preprocessing

**Figure 7:**
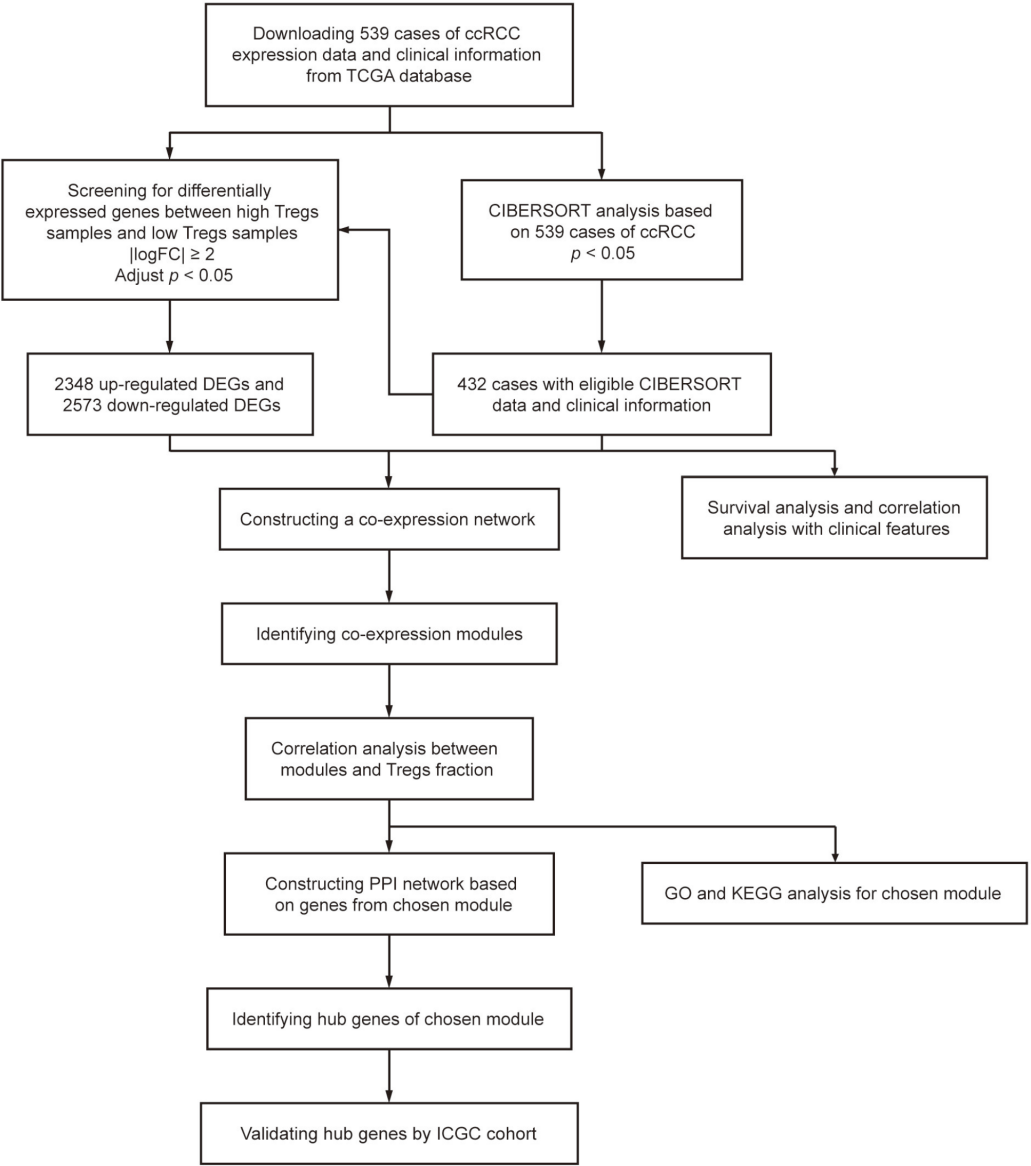
**Flowchart detailing the study design and samples at each stage of the analysis.**

A workflow of this study is shown in Figure 7. We downloaded mRNA expression profiles, including 539 cases of ccRCC samples, from the Cancer Genome Atlas (TCGA) database (https://portal.gdc.cancer.gov/) and cases without adequate clinical data were excluded. We used the CIBERSORT algorithm to evaluate tumor-infiltrating immune cells based on TGCA cohort. Meanwhile, survival analyses and correlation analyses with clinical features were conducted to evaluate the clinical significance of each type of immune cell. After screening the differentially expressed genes (DEGs) between samples with high and low proportions of Treg cells, WGCNA was conducted to determine the module relative to Treg cells. Gene Ontology (GO) analysis and pathway enrichment analysis were performed for functional annotation of selected modules. A protein–protein interaction (PPI) network was built and hub genes were selected according to the degree of connectivity. Meanwhile, an additional independent dataset of 231 cases of ccRCC samples and 70 normal kidney samples from the International Cancer Genomics Consortium (ICGC) database (https://dcc.icgc.org/) was used as a validation set.

### Evaluation of tumor-infiltrating immune cells

CIBERSORT is a deconvolution algorithm that uses a set of reference gene expression values (a “signature matrix” of 547 genes) considered a minimal representation for each cell type and, based on those values, infers cell type proportions in data from bulk tumor samples of mixed cell types using support vector regression [37]. Normalized gene expression data were used to infer the relative proportions of 22 types of infiltrating immune cells using the CIBERSORT algorithm. Briefly, gene expression datasets were prepared using standard annotation files and data uploaded to the CIBERSORT web portal (https://cibersort.stanford.edu/), with the algorithm run using the default signature matrix at 1,000 permutations. CIBERSORT derives a *P*-value for the deconvolution of each sample using Monte Carlo sampling, providing a measure of confidence in the results. From 539 cases of ccRCC samples analyzed, we selected 432 samples that met the requirements of CIBERSORT *P*-value <0.05.

### Differentially-expressed gene screening

The “limma” R package was used to screen the DEGs between samples with high and low proportions of Treg cells in TCGA cohort. Adjusted *P*-value<0.05 was considered significant statistically and |logFC|≥2 was set as the cut-off criterion for better accuracy and significance, as described previously [38, 39].

### Co-expression network construction

The expression profile data of DEGs was tested to check if they were good samples and genes. Then, we used the “WGCNA” R package to construct a scale-free co-expression network for the DEGs. The Pearson’s correlation matrices and average linkage method were both performed for all pair-wise genes. Then, a weighted adjacency matrix was constructed using a power function *a_mn_* = |*c_mn_*|^β^ (*c_mn_* = Pearson’s correlation between gene m and gene n; *a_mn_* = adjacency between gene m and gene n). β was a soft-thresholding parameter that could emphasize strong correlations between genes and penalize weak correlations. After choosing the power of β, the adjacency was transformed into a topological overlap matrix (TOM), which could measure the network connectivity of a gene defined as the sum of its adjacency with all other genes for network generation, and the corresponding dissimilarity (1-TOM) was calculated [40]. To classify genes with similar expression profiles into gene modules, average linkage hierarchical clustering was conducted according to the TOM-based dissimilarity measure with a minimum size (gene group) of 50 for the genes dendrogram.

### Identification of modules associated with the proportion of Treg cells

Two approaches were used to identify modules related to clinical traits of ccRCC. Firstly, gene significance (GS) was defined as the log10 transformation of the *P* value (GS = lg*P*) in the linear regression between gene expression and the clinical traits. In addition, module significance (MS) was defined as the average GS for all the genes in a module. Then, the module with the absolute MS ranked first among all the selected modules was considered as the one related to clinical traits. The module eigengene (ME) was considered as the major component in the principal component analysis for each gene module, and the expression patterns of all genes could be summarized into a single characteristic expression profile within a given module. In addition, we calculated the correlation between each ME and clinical traits to identify the relevant module. The module with the maximal absolute MS among all the selected modules was usually considered as the one related to clinical traits. Finally, the module highly correlated with certain clinical traits was selected for further analysis.

### GO and pathway enrichment analysis

Metascape (http://metascape.org/) is an online program providing a comprehensive set of functional annotation tools for investigators to understand the biological meaning of large lists of genes [41]. We uploaded genes into the selected module for GO analysis and pathway enrichment analysis. *P* <0.05 was considered statistically significant.

### PPI network and hub genes selection

Search Tool for the Retrieval of Interacting Genes (STRING) is a biological database for building PPI networks, providing a system-wide view of interactions between each member [42]. Genes from selected module were mapped to STRING to analyze their relationships with each other, and a combined score of >0.4 was set as the cut-off criterion, as described previously [43]. A PPI network was then established using Cytoscape software, which visually explores biomolecular interaction networks composed of proteins, genes, and other molecules. The plug-in Centiscape was used to search for the most important nodes in a network by calculating centrality parameters for each node [43]. Hub genes were selected from the PPI network according to the degree of connectivity.

### Survival analyses and ROC curve analyses of hub genes

A dataset including 231 cases of ccRCC samples and 70 normal kidney samples from the ICGC database was used to validate the different expression levels of the hub genes between ccRCC tissues and normal kidney tissues. Additionally, survival analyses for hub genes were performed. The hazard ratios (HRs) with 95% confidence intervals as well as log-rank *P*-values were calculated and displayed. Moreover, receiver operating characteristic (ROC) curves of hub genes were plotted and area under the curves (AUC) was calculated with the “ROC” R package to evaluate their capability to distinguish tumor and normal kidney tissue.

### Statistical analysis

Statistical analyses were conducted using SPSS version 22.0 software (SPSS, Chicago, IL, USA) and GraphPad Prism 5.0 (GraphPad Software, San Diego, CA, USA). The chi-square test for proportion was used to analyze the relationship between the fraction of Treg cells and clinicopathological parameters. Survival curves were plotted by the Kaplan-Meier method and compared with the log-rank test. Cox proportional hazard regression model was used to evaluate the influence of variables for overall survival. *P*<0.05 was considered statistically significant.
